# Contrasts in active transport behaviour across four countries: How do they translate into public health benefits?

**DOI:** 10.1016/j.ypmed.2015.02.009

**Published:** 2015-05

**Authors:** Thomas Götschi, Marko Tainio, Neil Maizlish, Tim Schwanen, Anna Goodman, James Woodcock

**Affiliations:** a Physical Activity and Health Unit, Institute of Epidemiology, Biostatistics and Prevention, University of Zurich, Zurich, Switzerland; b UKCRC Centre for Diet and Activity Research, MRC Epidemiology Unit, University of Cambridge School of Clinical Medicine, Institute of Metabolic Science, Cambridge, UK; c Systems Research Institute, Polish Academy of Sciences, Warsaw, Poland; d Berkeley, CA, USA; e School of Geography and the Environment, University of Oxford, Oxford, UK; f Faculty of Epidemiology and Population Health, London School of Hygiene and Tropical Medicine, London, UK

**Keywords:** Walking, Bicycling, Physical activity, Health impact assessment, Active travel

## Abstract

**Objective:**

Countries and regions vary substantially in transport related physical activity that people gain from walking and cycling and in how this varies by age and gender. This study aims to quantify the population health impacts of differences between four settings.

**Method:**

The Integrated Transport and Health Model (ITHIM) was used to estimate health impacts from changes to physical activity that would arise if adults in urban areas in England and Wales adopted travel patterns of Switzerland, the Netherlands, and California. The model was parameterised with data from travel surveys from each setting and estimated using Monte Carlo simulation. Two types of scenarios were created, one in which the total travel time budget was assumed to be fixed and one where total travel times varied.

**Results:**

Substantial population health benefits would accrue if people in England and Wales gained as much transport related physical activity as people in Switzerland or the Netherlands, whilst smaller but still considerable harms would occur if active travel fell to the level seen in California. The benefits from achieving the travel patterns of the high cycling Netherlands or high walking Switzerland were similar.

**Conclusion:**

Differences between high income countries in how people travel have important implications for population health.

## Highlights

• We model hypothetical health benefits from active travel for England & Wales.
• Scenarios reflect travel patterns from California, Switzerland and the Netherlands.
• Scenarios are based on travel surveys with realistic age and gender distributions.
• We explore several uncertainties using Monte Carlo simulation.
• We show impacts on cause-specific mortality and disability adjusted life years.

## Introduction

Regular physical activity provides a wide range of health benefits. Active travel (primarily walking and cycling) has gained attention from the transport and environmental sectors for its advantages as low-emission and space-efficient travel modes (Banister, 2008). Active travel is also increasingly recognized for its potential to contribute to overall physical activity (Craig et al., 2012; Dora, 1999). As active travel combines mobility and activity, it may offer a lower hurdle to be active than sports or other recreational activity. Nonetheless, steps to increase active travel have generally been hesitant, although some countries (e.g. the Netherlands, Switzerland, Germany or Denmark) have been more proactive than others (e.g. UK, USA). Health impact modelling is used to quantify effects of active travel on health outcomes in a specified population and as such can support informed decision making and cost-effective investment of limited resources.

In recent years, various methods to model health impacts of active travel have been developed. These typically compare benefits of physical activity with potential harms from injury risk and increased exposure to air pollution. When modelling substantial changes at the population level, such studies have overwhelmingly found large net benefits from active travel (de Hartog et al., 2010; Rabl and De Nazelle, 2012; Rojas-Rueda et al., 2011, 2013; Woodcock et al., 2013, 2014), although this may not apply in younger age groups when injury risks are high (Woodcock et al., 2014).

Typically health impact models of transport have used hypothetical scenarios with simplistic assumptions on changes in active travel (e.g. de Hartog et al., 2010; Gotschi, 2011; Grabow et al., 2011; Kahlmeier et al., 2011). Such studies may arguably struggle to realistically reflect travel behaviour, particularly in the context of advanced models which consider distributions of physical activity across age and gender. The objective of this study is therefore to create alternative scenarios using data from major travel surveys reflecting population-wide distributions of travel behaviour, in particular across age and gender. England and Wales (E&W) served as the reference scenario. To illustrate the potential range of the magnitude of health impacts from changes in active transport, comparison areas were chosen for exceptionally high or low levels of active transport, respectively. Specifically, the health impacts on the urban population of E&W were modelled, assuming shifts to travel patterns of Switzerland, the Netherlands, and California.

## Methods

### Travel survey data

Travel survey data were used from E&W and three comparison areas selected based on substantial contrasts in travel patterns, namely Switzerland for high levels of walking, the Netherlands for high levels of bicycling, and California for high levels of car usage. As such, they were used to inform hypothetical yet realistic scenarios for the population of E&W. Table 1 shows descriptive data of E&W and the three comparison areas.

Data on travel patterns were extracted from national travel surveys (Bundesamt für Statistik et al., 2007; Department of Transport, 2013; Federal Highway Administration, 2010; Ministerie van Verkeer en Waterstaat, 2010) (Supplementary Table A.1). To increase survey comparability, small communities of less than 10,000 inhabitants were excluded, and minimum trip duration was standardized to 3 min.

### Health impact modelling

Health impacts were modelled as changes in population health due to changes in active travel time (walking, cycling) in the E&W population. The model was estimated using Monte Carlo simulation in Analytica version 4.4. (www.lumina.com), running 50,000 iterations. The current E&W travel pattern was compared against the counterfactual scenarios in which E&W would adopt the travel patterns from Switzerland, the Netherlands or California, respectively. Travel patterns were modelled as changes in absolute terms (minutes of each mode), as well as relative terms (percent of total travel time of each mode). Travel behaviour was modelled as population wide distributions of travel times spent in different modes, stratified by sex and age groups for E&W and each comparison area. For all other variables, i.e. age distribution, background mortality and morbidity rates, age and sex-specific E&W data was used.

The study was conducted using a substantially improved and updated version of the Integrated Transport and Health Impact Modelling tool (ITHIM) (Woodcock, 2014), which now models variability and uncertainty of parameters using Monte Carlo simulation. Earlier versions were previously described elsewhere (Maizlish et al., 2013; Woodcock et al., 2013, 2014). ITHIM was used to model health benefits of physical activity using a range of non-linear dose–response functions specific to exposure domains (total physical activity, non-work physical activity, or physical activity from active travel) and outcomes (all cause mortality, morbidities). Because most previous health impact models of active travel found that associated risks are at least one order of magnitude smaller than benefits of physical activity when changes are modelled across all age groups (de Hartog et al., 2010; Rabl and De Nazelle, 2012; Rojas-Rueda et al., 2011), the approach to impact modelling presented here is only applied to impacts from physical activity.

Aggregation of background physical activities reflected intensity of specific activities, estimated in Metabolic Equivalents of Task (METs), as listed in the Compendium of Physical Activity (Ainsworth et al., 2011). Activities under 1.5 METs were excluded. METs were converted into marginal METs by subtracting 1 MET (intensity of being at rest). This approach only considers the activity over and above the metabolic activity at rest. Variation in METs for each activity was taken into account stochastically to generate distributions of METs within age and gender strata (Table 2).

Age (15 +) and gender specific data on walking, cycling, household work, sport and work included estimates of variability and were available from the health survey for England (Craig and Mindell, 2013). Background physical activity was assumed to remain unchanged throughout the different scenarios (Supplementary Table A.2).

Health benefits of physical activity were modelled using disease specific incidence and mortality of stroke, ischemic heart disease (IHD), other cardiovascular and circulatory diseases, type-2 diabetes, colon cancer, breast cancer, dementia and Alzheimer's disease, and depression. The doses were recalculated from Woodcock et al. (2009) as marginal MET/h week. See Table 3 for dose–response parameters. As part of sensitivity analysis, two alternative approaches to model impacts on all-cause mortality were applied, using relative risks from a systematic review by Woodcock et al. (2011) and a dose–response function presented in a recent large cohort study (Wen et al., 2011), respectively (Supplementary Table A.3).

A log-linear relationship was assumed between exposures and the health outcomes. Beyond this the exposure variables were transformed (using power transformations 0.25 to 1) (Sattelmair et al., 2011; Woodcock et al., 2011). Since the exact parameters of the non-linear dose–response function are unknown, these were stochastically allowed to vary across iterations of the model (see Supplementary Fig. B.1) and evaluated in sensitivity analyses.

Burden of disease data for the UK, including mortality rates as well as disability adjusted life years (DALYs), years of healthy life lost due to disability (YLDs) and years of life lost (YLLs), were obtained from the Global Burden of Disease (GBD) study 2010 (IHME, 2013) and adjusted to reflect E&W population size, and age and gender distribution. Supplementary Table A.4 presents size and age distribution and Supplementary Table A.5 the burden of disease for the study population.

Sensitivity of the model to selected parameters was illustrated with tornado plots (Table 2, Supplementary Table A.6 and Fig. B.2).

## Results

### Travel behaviour patterns

The three international comparison areas reveal substantial contrasts compared with E&W, both in terms of absolute travel times as well as relative distribution across travel modes assuming a constant travel time budget (Table 4). Overall, the data showed travel time to be highest in Switzerland at over 80 min per day, compared with fewer than 60 min in E&W. Californians drive the most, almost 1 h per day, compared with only around 35 min in E&W. The small differences for driving times between the different European settings reflect the fact that the Swiss and Dutch make fewer short but more longer trips by car, compared with E&W (data not shown).

The Dutch ranks the highest for cycling, at 12 min per day compared to only about 1 min per day in E&W. Levels of walking are highest in Switzerland at around 25 min per day.

**Fig. 1:**
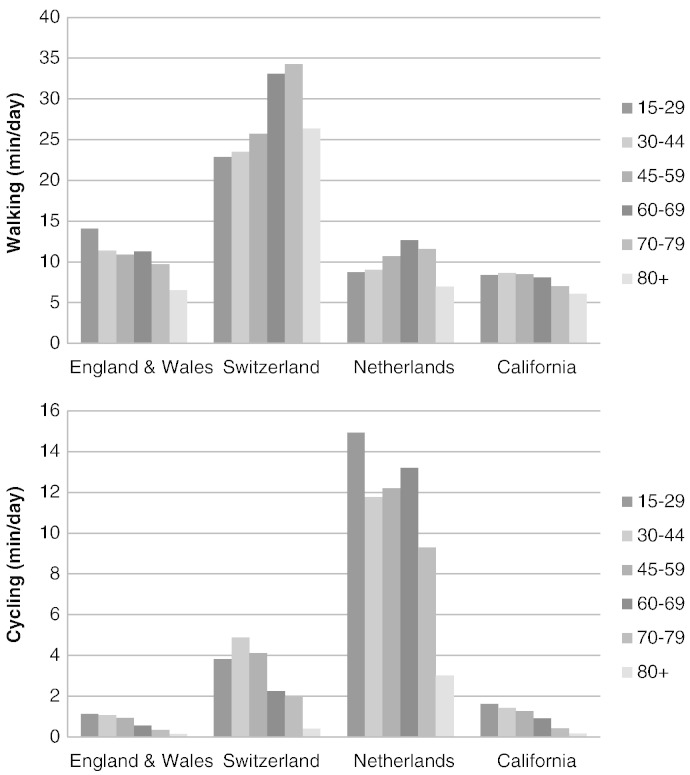
Age distribution of walking (top) and cycling (bottom) across E&W and three comparison areas^a^. ^a^The data is population based including people who did not travel. Communities < 10,000 and subjects < 16 years are excluded. E&W: N (persons) = 74,958, Source: Department of Transport, 2013. National Travel Survey statistics, 7/30/2013 ed. Switzerland: N (persons) = 40,473, Source: Mobilität in der Schweiz: Ergebnisse des Mikrozensus 2005 zum Verkehrsverhalten. Bundesamt fuer Statistik, Neuchatel. Netherlands N (persons) = 28,188, Source: Ministerie van Verkeer en Waterstaat, 2010. “Onderzoek Verplaatsingen in Nederland” (OViN). California N (persons) = 37,380, Source: Federal Highway Administration, 2010. National Household Travel Survey. US Department of Transportation.

Gender and age distributions of active travel are of interest both in planning — as indicators as to how well the environment supports active travel across the life course, and in health impact modelling (Table 4, Fig. 1 and Supplementary Fig. B.3). Walking is on balance slightly more common amongst women, whilst cycling shows a clear gender gap in favour of men in E&W and California, with almost three times lower values for women. In the Netherlands, where cycling is most popular and safest (Pucher and Buehler, 2008), there is no gender gap.

In E&W and California, walking levels are the highest amongst younger generations and decline with increasing age. In Switzerland and the Netherlands, walking levels increase with age and only drop notably in old age (80 +). For cycling, the decline with increasing age is dramatic in E&W, California and, albeit somewhat delayed, in Switzerland. In the Netherlands, cycling levels only decline after age 70 (Fig. 1).

### Health impacts

Estimated health impacts for adopting absolute travel times are presented in Table 5a, whilst Table 5b shows results for adopting proportions of travel time spent in different modes but keeping total travel time as at present. Every year, approximately 2.8 million DALYs are lost in E&W due to diseases associated with inactivity, i.e. cardiovascular diseases, breast and colon cancer, type-2 diabetes, dementia, and depression. This includes 167,000 deaths. Adopting time spent walking and cycling from Switzerland or the Netherlands, with substantially higher proportions of active travel, would prevent between 10,000 and 17,000 premature deaths, or between 150,000 to 250,000 DALYs, per year. On the other hand, adopting the travel pattern of California with less active travel would lead to additional 1700–3100 deaths, or 34,000–56,000 DALYs in E&W. Approximately half of the impacts on DALYs and deaths are attributable to IHD in males, and to IHD and stroke combined, in females. This reflects both the high incidence rates of IHD and stroke and their strong relationship with physical activity (Table 3).

Supplementary Table A.3 shows the sensitivity of impact estimates for deaths when applying different dose–response functions. In general, the health benefits and risks are two times larger when using the dose–response function for all-cause mortality presented by Woodcock et al. 2011 and three times larger when using the one from Wen et al. (2011), compared with the sum of deaths from the disease specific mortality model (Table 5a).

Tornado plot analyses showed that the model was most sensitive to assumptions on intensity of walking (METs), the shape of the dose–response function and the RR for stroke, IHD and other cardiovascular and circulatory diseases (Supplementary Fig. B.2). The relative contribution of these parameters to model uncertainty varied depending on the comparison being made.

## Discussion

Findings from this study imply that large, population level shifts in travel behaviour of E&W would translate into health impacts of significant magnitude. All else equal, adoption of high rates of active travel comparable to Switzerland or the Netherlands would result in the prevention of approximately 6–10% of all deaths caused by diseases associated with physical inactivity, and about 3–4% of all deaths due to all causes. Conversely, a shift towards somewhat lower levels of walking similar to California would result in up to 3000 additional premature deaths annually.

The comparisons also show that higher levels of active travel do not automatically correspond with less driving, which suggests that achieving high levels of active travel is not likely to be sufficient in itself to reduce carbon emissions unless there is also a policy to tackle longer car trips. Both the Swiss and Dutch spent similar time driving as people in E&W, but less so for short trips. Instead, there is a greater share of inter-urban car trips between more closely linked urban systems in these densely populated countries (Limtanakool et al., 2006).

### Study limitations and strengths

ITHIM is characterized by a number of strengths, compared with other similar models. In particular, it considers morbidity and not just deaths, population wide distributions of travel times, and health relevant parameters, such as gender, age, background disease rates and physical activity. It applies more realistic non-linear dose–response functions and estimates a set of different health measures.

The presented analysis uses empirical travel survey data to inform scenarios of shifts in travel patterns, which provide realistic population wide distributions of active travel by age and gender. For example, older age groups have higher health risks and therefore benefit more from relative risk reductions due to active travel. Typically, HIA rely on average effects. For example, the WHO HEAT tool (www.euro.who.int/HEAT) provides an approach that is conservative in excluding effects amongst older adults but may be optimistic in not differentiating between younger and middle-aged adults. In contrast, the presented approach considers realistic age and gender distributions in its scenarios. For example, increasing cycling to levels of the Netherlands means substantial increases in cycling in elderly and women (see Figs. 1 and B.3).

The two ways how travel patterns were applied are indicative of the range of resulting benefits. Achieving absolute travel times reflects more accurately our best estimates of how people in the different countries currently behave. However, increasing total travel times may be seen as undesirable and achieving changing the relative time spent might be a more appropriate policy target. It should also be noted that differences in total travel time between the settings may in part reflect differences in survey methods. Despite attempts to standardize, the comparison data entail inherent methodological differences (e.g. survey questions and periods) as well as local differences (e.g. land use and urban/rural mix).

To what extent and how such travel patterns could be adopted remains uncertain. Climate does not provide a good explanation of the differences in travel (Pucher and Buehler, 2006). Although the Netherlands has a favourable topography for cycling there are many flat areas in E&W without much cycling. The advent of electric assist bikes also offers the potential to reduce the burden of cycling in hillier areas. Probably of greater importance are high quality and safe infrastructure (Pucher and Dijkstra, 2003), as is in place in the Netherlands for cycling, synergies with public transport, as in Switzerland where public transport is fed by a huge number of walking trips (also see Table 4), and a generally supportive culture towards active travel (Heinen et al., 2010). Robust evidence on the beneficial effects on total physical activity of provision of traffic free walking and cycling routes is emerging from the UK (Goodman et al., 2014), and studies have shown a positive increase in cycling following city level programmes in England (Goodman et al., 2013). However, overall quantitative effects of specific policies remain poorly understood (Goodman et al., 2014; Pucher et al., 2010), and depend on cultural context that may require adapted local approaches (Aldred and Jungnickel, 2014). Well documented is the correlation between levels of active transport and traffic safety (Elvik, 2009; Jacobsen, 2003; Pucher and Dijkstra, 2003). Perceived risks are a major barrier to cycling and to an extent to walking, and increasing objective and subjective safety in a manner that does not detract from mode convenience should be considered by all policies to promote active travel.

Interestingly the benefits in E&W from adopting travel patterns from the Netherlands or Switzerland were similar. Based on absolute travel times, benefits from Swiss travel (mainly walking) were greater in part because the Swiss spend more time travelling than people in the other settings. If instead we assume that the English and Welsh travel time budget remains constant but is proportioned out differently, then the benefits from Dutch travel were greater.

Several methodological considerations are of importance when interpreting the presented health impacts. Because the dose–response relationship is non-linear, less active individuals benefit more from increasing physical activity than those already active. The calculation also assumes no effect on other physical activities from shifting travel patterns. As shown, the dose–response function is quite influential, but there is no scientific consensus on its exact shape or how it may vary by disease (Samitz et al., 2011; Sattelmair et al., 2011; Woodcock et al., 2011). A power transformation of 0.25 attributes a large part of benefits to moving from no activity to low levels of activity, whereas a closer to linear shape distributes benefits more evenly across a wider exposure range.

The presented analysis is limited by its focus on benefits from physical activity. Increases in active travel could also lead to increased risk for crashes and increased exposure to air pollution. Previous studies indicate that if a population wide change in behaviour is achieved then benefits from physical activity by far outweigh these risks either at population or individual level (de Hartog et al., 2010; Woodcock et al., 2009).

This study points to several research needs. Handling non-linear dose–response functions and non-normal distributions of key parameters remain challenges. Meta-analyses of active travel related relative risks and dose–response functions are needed. Travel surveys combined with objective measurements should collect improved data including intensity of walking and cycling, ideally complemented by items on overall physical activity. Ultimately, policy evaluations should collect adequate data to link findings from health impact modelling directly to prior investments made to achieve shifts towards healthier travel patterns.

## Conclusions

International surveys can be used to inform scenario calculations for increasingly sophisticated health impact models of active travel. Travel survey data would be even more valuable, if items on physical activity were included and international calibration would increase feasibility of such comparisons.

If E&W adopted walking and cycling patterns of Switzerland and the Netherlands this could be expected to confer major health benefits. As such, the presented findings provide strong support for investments in efforts to increase levels of active travel.

## Conflict of interest statement

The authors declare that there are no conflicts of interest.

## Figures and Tables

**Table 1:** Descriptive data on E&W and three comparison areas.

	England & Wales (study area)	Switzerland	Netherlands	California
Population (million)	55.6 (2010)^a^	8.0 (2011)	16.8 (2014)	38.3 (2013)
Area	151,036 km^2^	41,285 km^2^	41,543 km^2^	423,970 km^2^
Population density b	370/km^2^	188/km^2^	405/km^2^	95/km^2^
Gross domestic product (GDP)/capita ($)	37 k (UK)	54 k	42 k	46 k (USA)
Cars per household	1.2 (2011)^b^	1.15 (2010)^c^	1.0 (2005)^d^	2.2 (2010)^e^
Share of trips by walking and cycling	26% (UK, 2008)^f^	50% (2010)^d^	51% (2008)^g^	23% (2012)^h^

*Sources: (**Wikipedia, 2014**), if not otherwise stated. See footnotes.*

a http://www.ons.gov.uk/ons/rel/pop-estimate/population-estimates-for-uk--england-and-wales--scotland-and-northern-ireland/mid-2001-to-mid-2010-revised/index.html

b http://www.ons.gov.uk/ons/rel/census/2011-census/key-statistics-for-local-authorities-in-england-and-wales/index.html

c Bundesamt für Statistik and Bundesamt für Raumentwicklung, 2012. Mobilität in der Schweiz: Ergebnisse des Mikrozensus Mobilität und Verkehr 2010. BfS, ARE, Neuchâtel.

d http://www.cbs.nl/en-GB/menu/themas/dossiers/nederland-regionaal/publicaties/artikelen/archief/2007/2007-2127-wm.htm

e http://www.clrsearch.com/Sacramento-Demographics/CA/Number-of-Vehicles-per-Household

f https://www.gov.uk/government/collections/national-travel-survey-statistics

g Ministerie van Verkeer en Waterstaat, 2010. “Onderzoek Verplaatsingen in Nederland” (OViN).

h Federal Highway Administration, 2010. National Household Travel Survey. US Department of Transportation.

**Table 2:** Intensity parameters used to derive background physical activity and physical activity from active travel. (Source: Compendium of Physical Activities (https://sites.google.com/site/compendiumofphysicalactivities/) (Ainsworth et al., 2011)).

Physical activity	Estimate (marginal METs)	Distribution	Description in compendium of physical activities (Ainsworth et al., 2011)
Walking	Mean: 2.5	Lognormal (stddev:1.6)	Mean MET (3.5) refers to METs for “walking for pleasure.” Variability by author judgement.
Cycling	Median: 5.8	LogNormal (gsdev:1.3)	Median MET (6.8) refers to METs for “bicycling, to/from work, self selected pace.” Variability by author judgement.
Household work	Median: 3.5	LogNormal (gsdev:1.5)	Median MET (4.5) refers to METs for “polishing floors, standing, walking slowly, using electric polishing machine.” Variability by author judgement.
Sports	Median:5	LogNormal (gsdev:1.5)	Median MET (6.0) refers to METs for “volleyball, competitive, in gymnasium.” Variability by author judgement.

^a^MET is defined as the ratio of activity specific metabolic rate to a standard resting metabolic rate of 1.0 (1.0 kcal/(kg ∗ h) or 4.184 kJ/(kg ∗ h)) ((Ainsworth et al., 2000)). Marginal METs refer to the intensity of activity over and above the resting metabolic rate. Marginal METh/wk are calculated as (MET rate - 1) * hours of activity.

**Table 3:** Dose–response parameters for different diseases.

Disease	RR (mean (std))^a^	Corresponding exposure marginal MET h/week)^b^	Reference
Stroke; ischemic heart disease (IHD); other cardiovascular and circulatory diseases	0.84 (0.03)	5.4	Hamer and Chida (2008)
Type-2 diabetes	0.83 (0.04)	5.6	Jeon et al. (2007)
Colon cancer	Men: 0.80 (0.08); women: 0.86 (0.06)	Men 24.1; women 23.3	Harriss et al. (2009)
Breast cancer	0.94 (0.01)	3.5	Monninkhof et al. (2007)
Dementia and Alzheimer's disease	0.72 (0.07)	24.5	Hamer and Chida (2009)
Depression	0.96 (0.02)	0.8	Paffenbarger et al. (1994)

a Means and standard deviations are based on a normal distribution.

b E.g. RR of 0.84 moving from no activity to 5.4 marginal MET h/week.

**Table 4:** Daily average^a^ travel times (minutes (% of total travel time)) by mode in E&W and three comparison areas.

	England & Wales	Switzerland	Netherlands	California
*Male*				
Walk	10.7 (17.6%)	23.4 (25.4%)	9.3 (12.2%)	7.5 (9.4%)
Cycle	1.4 (2.3%)	4.5 (4.9%)	12.7 (16.6%)	2.0 (2.5%)
Public transport	7.1 (11.7%)	13.9 (15.1%)	8.7 (11.3%)	5.9 (7.4%)
Car	38.1 (62.5%)	44.7 (48.5%)	40.7 (53.2%)	61.8 (77.5%)
Other	3.6 (6.0%)	5.6 (6.1%)	5.1 (6.7%)	2.5 (3.1%)
Total	61.0 (100.0%)	92.1 (100.0%)	76.4 (100.0%)	79.8 (100.0%)

*Female*				
Walk	12.4 (22.3%)	28.5 (36.1%)	10.4 (16.6%)	8.9 (12.1%)
Cycle	0.4 (0.7%)	2.9 (3.7%)	11.7 (18.7%)	0.5 (0.7%)
Public transport	7.8 (14.1%)	14.0 (17.7%)	8.7 (13.9%)	6.3 (8.5%)
Car	34.1 (61.5%)	31.1 (39.4%)	28.6 (45.4%)	56.8 (77.2%)
Other	0.8 (1.4%)	2.4 (3.1%)	3.4 (5.5%)	1.1 (1.5%)
Total	55.4 (100.0%)	78.9 (100.0%)	62.9 (100.0%)	73.6 (100.0%)

a The data is population based including people who did not travel. Communities < 10,000 and subjects < 16 years are excluded.

**Table 5a:** Changes in DALYs (left) and deaths (right) in England & Wales (age 15+) if adopting travel patterns from comparison areas (based on absolute travel times) (Median (95% credible interval)). (See Table 5b for adopting relative travel time distribution across modes).

	Baseline DALYs in England & Wales	Adopted travel pattern from			Baseline deaths in England & Wales	Adopted travel pattern from		
		Switzerland	Netherlands	California		Switzerland	Netherlands	California
*Males*								
Stroke	201,995	*− 21,310 (− 33,140 to − 11,870)*	*− 18,090 (− 29,380 to − 9480)*	*3365 (2026 to 4851)*	14,047	*− 1525 (− 2344 to − 862)*	*− 1121 (− 1795 to − 600)*	*226 (134 to 335)*
Ischemic heart disease	620,598	*− 63,400 (− 99,070 to − 35,100)*	*− 57,950 (− 94,640 to − 30,150)*	*10,220 (6150 to 14,710)*	38,329	*− 4,122 (− 6376 to − 2312)*	*− 3293 (− 5314 to − 1743)*	*629 (377 to 917)*
Other cardiovascular and circulatory diseases	171,381	*− 17,770 (− 27,730 to − 9856)*	*− 15,800 (− 25,780 to − 8224)*	*2877 (1735 to 4134)*	10,388	*− 1127 (− 1741 to − 633)*	*− 875 (− 1408 to − 465)*	*171 (102 to 250)*
Type-2 diabetes	64,498	*− 6546 (− 10,630 to − 3177)*	*− 6145 (− 10,380 to − 2838)*	*1091 (546 to 1707)*	1850	*− 207 (− 333 to − 102)*	*− 164 (− 272 to − 77)*	*32 (16 to 50)*
Colon cancer	76,689	*− 3884 (− 7299 to − 738)*	*− 3774 (− 7126 to − 722)*	*530 (92 to 1073)*	4375	*− 238 (− 446 to − 46)*	*− 208 (− 389 to − 40)*	*32 (5 to 65)*
Breast cancer	–	–	–	*–*	*–*	*–*	*–*	*–*
Dementia and Alzheimer's disease	89,714	*− 7163 (− 11,150 to − 3428)*	*− 5571 (− 8655 to − 2671)*	*918 (380 to 1682)*	5366	*− 431 (− 672 to − 206)*	*− 303 (− 471 to − 146)*	*51 (20 to 98)*
Depression	137,704	*− 7715 (− 20,770 to − 1319)*	*− 8929 (− 24,180 to − 1416)*	*1168 (225 to 3109)*	–	–	–	–
Total	1,362,578	*− 127,800 (− 196,500 to − 75,850)*	*− 116,300 (− 188,800 to − 63,700)*	*20,160 (13,500 to 27,460)*	74,357	*− 7651 (− 11,420 to − 4611)*	*− 5963 (− 9290 to − 3400)*	*1140 (718 to 1637)*

*Females*								
Stroke	226,967	*− 24,630 (− 37,100 to − 14,230)*	*− 15,950 (− 23,920 to − 9249)*	*2421 (1438 to 3568)*	22,603	*− 2506 (− 3711 to − 1472)*	*− 1191 (− 1699 to − 717)*	*154 (76 to 263)*
Ischemic heart disease	337,852	*− 36,580 (− 55,360 to − 21,050)*	*− 25,730 (− 38,990 to − 14,790)*	*3933 (2368 to 5685)*	31,224	*− 3456 (− 5135 to − 2023)*	*− 1786 (− 2574 to − 1066)*	*239 (128 to 389)*
Other cardiovascular and circulatory diseases	149,553	*− 16,040 (− 24,320 to − 9215)*	*− 11,440 (− 17,420 to − 6537)*	*1811 (1093 to 2606)*	12,442	*− 1375 (− 2043 to − 805)*	*− 710 (− 1024 to − 424)*	*96 (52 to 156)*
Type-2 diabetes	56,938	*− 6199 (− 9903 to − 3067)*	*− 5075 (− 8239 to − 2471)*	*829 (423 to 1260)*	1977	*− 226 (− 354 to − 115)*	*− 125 (− 191 to − 64)*	*17 (8 to 29)*
Colon cancer	58,147	*− 2140 (− 4100 to − 294)*	*− 1804 (− 3470 to − 248)*	*238 (31 to 484)*	3,850	*− 151 (− 287 to − 21)*	*− 99 (− 189 to − 14)*	*12 (1 to 26)*
Breast cancer	201,757	*− 8966 (− 17,870 to − 3637)*	*− 8514 (− 17,530 to − 3262)*	*1078 (527 to 1877)*	9096	*− 429 (− 819 to − 185)*	*− 336 (− 658 to − 139)*	*40 (21 to 65)*
Dementia and Alzheimer's disease	151,156	*− 12,450 (− 19,590 to − 5902)*	*− 6693 (− 10,770 to − 3114)*	*718 (247 to 1577)*	11,873	*− 1000 (− 1586 to − 471)*	*− 433 (− 729 to − 192)*	*34 (6 to 95)*
Depression	230,906	*− 16,030 (− 42,830 to − 2829)*	*− 17,010 (− 45,680 to − 2871)*	*2899 (563 to 7611)*	–	–	–	–
Total	1,413,276	*− 123,000 (− 188,600 to − 76,930)*	*− 92,230 (− 148,900 to − 55,040)*	*13,930 (9748 to 18,840)*	93,067	*− 9144 (− 12,910 to − 5947)*	*− 4678 (− 6417 to − 3121)*	*592 (343 to 948)*
Total (males + females)	2,775,854	*−*250,800 (*−*384,100 to *−*153,300)	*−*208,500 (*−*336,900 to *−*119,200)	34,090 (23,630 to 45,210)	167,423	*−*16,800 (*−*24,320 to *−*10,580)	*−*10,640 (*−*15,660 to *−*6595)	1732 (1072 to 2575)

**Table 5b:** Changes in DALYs (left) and deaths (right) in England & Wales (age 15+) if adopting travel patterns from comparison areas (based on relative travel time distribution by mode applied to constant E&W total travel time) (Median (95% credible interval)). (See Table 5a for adopting absolute travel times for each mode).

	Baseline DALYs in England & Wales	Adopted travel pattern from			Baseline deaths in England & Wales	Adopted travel pattern from		
		Switzerland	Netherlands	California		Switzerland	Netherlands	California
Males								
Stroke	201,995	*− 13,190 (− 19,130 to − 7823)*	*− 16,070 (− 25,820 to − 8594)*	*5070 (3087 to 7126)*	14,047	*− 941 (− 1347 to − 564)*	*− 1042 (− 1656 to − 565)*	*343 (210 to 478)*
Ischemic heart disease	620,598	*− 39,510 (− 57,760 to − 23,330)*	*− 50,490 (− 81,480 to − 26,890)*	*15,570 (9450 to 21,950)*	38,329	*− 2549 (− 3676 to − 1518)*	*− 2987 (− 4773 to − 1608)*	*950 (581 to 1331)*
Other cardiovascular and circulatory diseases	171,381	*− 11,010 (− 16,050 to − 6504)*	*− 13,840 (− 22,320 to − 7370)*	*4342 (2637 to 6116)*	10,388	*− 696 (− 1001 to − 415)*	*− 799 (− 1276 to − 431)*	*258 (158 to 360)*
Type-2 diabetes	64,498	*− 4111 (− 6363 to − 2087)*	*− 5291 (− 8858 to − 2484)*	*1676 (865 to 2525)*	1850	*− 128 (− 196 to − 66)*	*− 149 (− 246 to − 71)*	*48 (25 to 72)*
Colon cancer	76,689	*− 2260 (− 4302 to − 419)*	*− 3217 (− 6076 to − 612)*	*863 (156 to 1667)*	4375	*− 139 (− 265 to − 25)*	*− 183 (− 343 to − 35)*	*51 (9 to 99)*
Breast cancer	–	–	–	–	–	–	–	–
Dementia and Alzheimer's disease	89,714	*− 4251 (− 6870 to − 1961)*	*− 5127 (− 7984 to − 2452)*	*1488 (667 to 2483)*	5366	*− 258 (-421 to − 118)*	*− 288 (− 448 to − 138)*	*86 (38 to 146)*
Depression	137,704	*− 4207 (− 11,340 to − 762)*	*− 6883 (− 19,010 to − 1102)*	*2216 (391 to 6105)*	–	*–*	*–*	*–*
Total	1,362,578	*− 78,010 (− 111,000 to − 50,330)*	*− 100,500 (− 160,600 to − 56,720)*	*31,540 (20,950 to 43,400)*	74,357	*− 4702 (− 6533 to − 3027)*	*− 5466 (− 8429 to − 3166)*	*1725 (1124 to 2343)*

Females								
Stroke	226,967	*− 15,110 (− 21,250 to − 9184)*	*− 16,030 (− 23,980 to − 9307)*	*4489 (2722 to 6342)*	22,603	*− 1463 (− 2037 to − 899)*	*− 1319 (− 1905 to − 786)*	*351 (215 to 489)*
Ischemic heart disease	337,852	*− 22,790 (− 32,170 to − 13,810)*	*− 25,330 (− 38,210 to − 14,610)*	*7004 (4233 to 9934)*	31,224	*− 2043 (− 2849 to − 1254)*	*− 1924 (− 2802 to − 1143)*	*511 (313 to 714)*
Other cardiovascular and circulatory diseases	149,553	*− 10,020 (− 14,170 to − 6,067)*	*− 11,200 (− 16,980 to − 6437)*	*3182 (1921 to 4518)*	12,442	*− 813 (− 1133 to − 499)*	*− 765 (− 1114 to − 454)*	*204 (125 to 286)*
Type*-*2 diabetes	56,938	*− 4022 (− 6140 to − 2066)*	*− 4817 (− 7781 to − 2359)*	*1389 (710 to 2122)*	1977	*− 136 (− 203 to -71)*	*− 132 (− 204 to − 67)*	*35 (18 to 53)*
Colon cancer	58,147	*− 1299 (− 2517 to − 175)*	*− 1688 (− 3239 to − 232)*	*429 (57 to 834)*	3850	*− 88 (− 173 to − 12)*	*− 99 (− 189 to − 13)*	*25 (3 to 48)*
Breast cancer	201,757	*− 5507 (− 10,390 to − 2430)*	*− 7753 (− 15,780 to − 3031)*	*1955 (870 to 3673)*	9096	*− 252 (− 446 to − 120)*	*− 319 (− 616 to − 135)*	*79 (37 to 143)*
Dementia and Alzheimer's disease	151,156	*− 7166 (− 12,210 to − 3141)*	*− 7139 (− 11,480 to − 3324)*	*1783 (795 to 3004)*	11,873	*− 560 (− 980 to − 239)*	*− 500 (− 826 to − 225)*	*117 (52 to 202)*
Depression	230,906	*− 10,110 (− 26,970 to − 1859)*	*− 14,870 (− 40,170 to − 2540)*	*4781 (875 to 12,990)*	–	*–*	*–*	*–*
Total	1,413,276	*− 76,240 (− 107,500 to − 51,900)*	*− 88,860 (− 140,900 to − 54,150)*	*24,940 (16,520 to 37,260)*	93,067	*− 5349 (− 7090 to − 3656)*	*− 5047 (− 6970 to − 3348)*	*1320 (899 to 1752)*
Total (males + females)	2,775,854	*−*154,700 (*−*218,400 to *−*102,700)	*−*189,800 (*−*301,100 to *−*111,500)	56,070 (37,520 to 79,180)	167,423	*−*10,060 (*−*13,550 to *−*6712)	*−*10,460 (*−*15,280 to *−*6539)	3092 (2058 to 4159)
