# Supplementary material for: Contrasts in active transport behaviour across four countries: How do they translate into public health benefits?
Source: Prev Med. 2015 May;74:42–8. doi: 10.1016/j.ypmed.2015.02.009 (PMC4456468; doi:10.1016/j.ypmed.2015.02.009)
Supplement: Supplementary file 1 — Supplementary tables and figures. [file mmc1.docx]

Appendix A: Supplementary Tables

Table A.1: Overview of travel surveys used

| **Survey** | **England & Wales (urban)** | **California** | **Netherlands (urban)** | **Switzerland (urban)** |
| --- | --- | --- | --- | --- |
| **Name** | National Travel Survey | National Household Travel Survey | Onderzoek Verplaatsingen in Nederland | Mikrozensus Verkehr |
| **Frequency** | Annually | Approx. every 7 years | Annually | Every five years |
| **Year(s) analysed** | 2002-2008 | 2009 | 2005 | 2005 |
| **Survey size (subjects)** | 20,000 per year | 40,280 (2009) | 35,000 (2005) | 33,390 (2005) |
| **Survey method** | Face to face interviews + 1 week self-completed travel diary | Computer-assisted telephone interviewing (CATI) + 1-day travel diary | Computer-assisted telephone interviewing (CATI) + 1-day travel diary | Computer-assisted telephone interviewing (CATI) + 1-day travel diary |
| **Length of travel diary** | 7 days but walking trips <1 mile only on 1 day | 1 day travel diary | 1 day travel diary | 1 day travel diary |
| **Sample restrictions** | Excluded communities of less than 10k inhabitants and rural areas | Restricted to urban areas | Excluded communities categorized as barely urban or not urban. | Excluded communities of less than 10k inhabitants and rural areas.  Excluded trips less than 3 min and walking trips >10km. |
| **Age range analyzed** | 15+ | All | All | 6+ |
| **Time & distance estimated by** | Self-reported time & distance | Self-reported time & distance | Self-reported time & distance | Self-reported time & distance |
| **Data source reference** | ([Department of Transport, 2013](#_ENREF_5)) | ([Federal Highway Administration, 2010](#_ENREF_6)) | ([Ministerie van Verkeer en Waterstaat, 2010](#_ENREF_8)) | ([Bundesamt für Statistik et al., 2007](#_ENREF_3)) |

**Table A.2:** Background physical activity (mean marginal METh/week)^a^ due to non-travel related activities in England & Wales. (Source: Health Survey for England ([Craig and Mindell, 2013](#_ENREF_4))).

| **Age group** | **Non-travel related physical activity (marginal METhs/week)** | | | | **Non-travel, non-work related physical activity (marginal METhs/week)** | | | |
| --- | --- | --- | --- | --- | --- | --- | --- | --- |
|  | Male | | Female | | Male | | Female | |
|  | Mean | SD | Mean | SD | Mean | SD | Mean | SD |
| 15-29 | 7.8 | 7.54 | 5.2 | 6.44 | 4.4 | 4.92 | 2.8 | 3.56 |
| 30-44 | 7.9 | 8.05 | 5.3 | 5.92 | 2.8 | 3.19 | 2.6 | 3.65 |
| 45-59 | 7.7 | 7.81 | 6.3 | 6.98 | 2.8 | 3.52 | 2.6 | 4.29 |
| 60-69 | 4.7 | 6.00 | 3.8 | 4.80 | 2.8 | 3.63 | 2.6 | 3.46 |
| 70-79 | 3.4 | 5.46 | 2.1 | 2.80 | 3.1 | 5.29 | 1.9 | 2.66 |
| 80+ | 2.2 | 3.69 | 1.2 | 1.77 | 1.7 | 2.18 | 1.2 | 1.74 |

^a^ MET is defined as the ratio of activity specific metabolic rate to a standard resting metabolic rate of 1.0 (1.0 kcal/(kg*h) or 4.184 kJ/(kg*h)) (([Ainsworth et al., 2000](#_ENREF_2))). Marginal METh/week refers to physical activity in addition to metabolic activity of being at rest and is calculated as MET rate – 1 * hours per week for each activity or METh/week – hours per week of activity.

| **Table A.3:** Sensitivity of impacts on annual deaths per 100,000 in England & Wales if adopting other travel patterns (absolute travel times), depending on different exposure-response functions. (Median (95% credible interval)). |
| --- |

| Males | Adopted travel pattern from | | |
| --- | --- | --- | --- |
|  | Switzerland | Netherlands | California |
| Sum of disease-specific calculations (Table 4a) | *-7651 (-11,420 to -4611)* | *-5963 (-9,290 to -3400)* | *1140 (718 to 1637)* |
| All-cause mortality from Woodcock et al. ([2009](#_ENREF_10)) | *-16,310 (-23,400 to -10,570)* | *-13,530 (-20,230 to -8322)* | *2,084 (1604 to 2584)* |
| All-cause mortality from Wen et al. ([2011](#_ENREF_9))^a^ | *-23,700* | *-18,740* | *2991* |
| Females | Adopted travel pattern from | | |
|  | Switzerland | Netherlands | California |
| Sum of disease-specific calculations (Table 4a) | *-9144 (-12,910 to -5947)* | *-4678 (-6417 to -3121)* | *592 (343 to 948)* |
| All-cause mortality from Woodcock et al. ([2009](#_ENREF_10)) | *-18,090 (-24,180 to -12,880)* | *-10,340 (-13,610 to -7477)* | *1112 (784 to 1585)* |
| All-cause mortality from Wen et al. ([2011](#_ENREF_9))^a^ | *-26,950* | *-15,270* | *1,297* |

^a^ Credible intervals not calculated

**Table A.4:** Study population in England & Wales. The analysis includes only population living in communities with over 10,000 inhabitants. (Source: Census 2011, www.ons.gov.uk)

|  | **Male** | **Female** | **Total** |
| --- | --- | --- | --- |
| 15-29 | 4,524,300 | 4,524,300 | 9,048,600 |
| 30-44 | 5,232,650 | 5,232,650 | 10,465,300 |
| 45-59 | 4,053,133 | 4,218,567 | 8,271,700 |
| 60-69 | 2,127,335 | 2,214,165 | 4,341,500 |
| 70-79 | 1,450,518 | 1,702,782 | 3,153,300 |
| 80+ | 625,633 | 1,065,267 | 1,690,900 |
| Total | 18,013,569 | 18,957,731 | 36,971,300 |

**Table A.5:** Annual background DALY, YLD, YLL and deaths in E&W. Data was calculated from the Global Burden of Disease (GBD) study 2010 ([IHME, 2013](#_ENREF_7)) (http://ghdx.healthdata.org/record/united-kingdom-global-burden-disease-study-2010-gbd-2010-results-1990-2010).

| **Deaths, Males** | **15-29** | **30-44** | **45-59** | **60-69** | **70-79** | **80+** | **Sum** |
| --- | --- | --- | --- | --- | --- | --- | --- |
| **Stroke** | 32 | 201 | 654 | 1,320 | 3,913 | 7,928 | 14,047 |
| **Ischemic heart disease** | 42 | 729 | 3,724 | 6,372 | 11,560 | 15,901 | 38,329 |
| **Other cardiovascular and circulatory diseases** | 36 | 212 | 683 | 1,428 | 3,246 | 4,784 | 10,388 |
| **Type-2 diabetes** | 0 | 65 | 155 | 265 | 590 | 775 | 1,850 |
| **Colon cancer** | 10 | 80 | 455 | 995 | 1,508 | 1,327 | 4,375 |
| **Breast cancer** | 0 | 0 | 0 | 0 | 0 | 0 | 0 |
| **Dementia and Alzheimer’s disease** | 2 | 5 | 45 | 239 | 1,284 | 3,791 | 5,366 |
| **Depression** | 0 | 0 | 0 | 0 | 0 | 0 | 0 |
| **All cause mortality** | 2,832 | 7,053 | 17,451 | 28,871 | 53,508 | 74,222 | 183,938 |
|  |  |  |  |  |  |  |  |
| **DALY, Males** | **15-29** | **30-44** | **45-59** | **60-69** | **70-79** | **80+** | **Sum** |
| **Stroke** | 2,340 | 10,979 | 26,314 | 36,957 | 66,625 | 58,781 | 201,995 |
| **Ischemic heart disease** | 3,271 | 37,800 | 134,160 | 157,312 | 178,213 | 109,843 | 620,598 |
| **Other cardiovascular and circulatory diseases** | 2,815 | 11,892 | 27,178 | 38,446 | 54,738 | 36,310 | 171,381 |
| **Type-2 diabetes** | 39 | 9,006 | 15,914 | 14,996 | 16,142 | 8,402 | 64,498 |
| **Colon cancer** | 649 | 3,855 | 15,525 | 23,825 | 23,332 | 9,502 | 76,689 |
| **Breast cancer** | 0 | 0 | 0 | 0 | 0 | 0 | 0 |
| **Dementia and Alzheimer’s disease** | 153 | 302 | 4,112 | 10,380 | 29,731 | 45,036 | 89,714 |
| **Depression** | 30,562 | 43,426 | 35,258 | 15,530 | 8,995 | 3,933 | 137,704 |
| **All cause mortality** | 622,693 | 986,311 | 1,209,942 | 1,078,192 | 1,119,562 | 667,565 | 5,684,264 |
|  |  |  |  |  |  |  |  |
| **YLD, Males** | **15-29** | **30-44** | **45-59** | **60-69** | **70-79** | **80+** | **Sum** |
| **Stroke** | 374 | 1,497 | 4,349 | 6,841 | 10,772 | 6,960 | 30,794 |
| **Ischemic heart disease** | 704 | 3,987 | 9,736 | 10,633 | 10,510 | 5,898 | 41,467 |
| **Other cardiovascular and circulatory diseases** | 612 | 1,876 | 4,381 | 5,827 | 7,875 | 5,041 | 25,612 |
| **Type-2 diabetes** | 20 | 5,894 | 10,673 | 8,925 | 7,609 | 3,337 | 36,457 |
| **Colon cancer** | 12 | 101 | 508 | 953 | 1,227 | 830 | 3,631 |
| **Breast cancer** | 0 | 0 | 0 | 0 | 0 | 0 | 0 |
| **Dementia and Alzheimer’s disease** | 0 | 43 | 2,659 | 5,012 | 11,728 | 20,256 | 39,698 |
| **Depression** | 30,562 | 43,426 | 35,258 | 15,530 | 8,995 | 3,933 | 137,704 |
| **All cause mortality** | 444,560 | 649,023 | 623,988 | 414,484 | 343,555 | 182,382 | 2,657,992 |
|  |  |  |  |  |  |  |  |
| **YLL, Males** | **15-29** | **30-44** | **45-59** | **60-69** | **70-79** | **80+** | **Sum** |
| **Stroke** | 1,966 | 9,482 | 21,965 | 30,115 | 55,853 | 51,821 | 171,202 |
| **Ischemic heart disease** | 2,567 | 33,813 | 124,424 | 146,680 | 167,703 | 103,946 | 579,131 |
| **Other cardiovascular and circulatory diseases** | 2,203 | 10,017 | 22,798 | 32,619 | 46,863 | 31,269 | 145,769 |
| **Type-2 diabetes** | 19 | 3,112 | 5,241 | 6,070 | 8,533 | 5,065 | 28,041 |
| **Colon cancer** | 637 | 3,755 | 15,018 | 22,872 | 22,104 | 8,672 | 73,058 |
| **Breast cancer** | 0 | 0 | 0 | 0 | 0 | 0 | 0 |
| **Dementia and Alzheimer’s disease** | 153 | 259 | 1,453 | 5,368 | 18,003 | 24,779 | 50,015 |
| **Depression** | 0 | 0 | 0 | 0 | 0 | 0 | 0 |
| **All cause mortality** | 178,133 | 337,288 | 585,953 | 663,709 | 776,008 | 485,181 | 3,026,272 |

| **Deaths, Females** | **15-29** | **30-44** | **45-59** | **60-69** | **70-79** | **80+** | **Sum** |
| --- | --- | --- | --- | --- | --- | --- | --- |
| **Stroke** | 22 | 160 | 532 | 1,020 | 4,015 | 16,855 | 22,603 |
| **Ischemic heart disease** | 14 | 165 | 895 | 2,252 | 6,925 | 20,974 | 31,224 |
| **Other cardiovascular and circulatory diseases** | 27 | 130 | 384 | 831 | 2,701 | 8,369 | 12,442 |
| **Type-2 diabetes** | 0 | 36 | 92 | 162 | 474 | 1,213 | 1,977 |
| **Colon cancer** | 8 | 70 | 350 | 616 | 1,120 | 1,685 | 3,850 |
| **Breast cancer** | 17 | 594 | 1,936 | 1,913 | 2,046 | 2,591 | 9,096 |
| **Dementia and Alzheimer’s disease** | 2 | 3 | 44 | 226 | 1,563 | 10,034 | 11,873 |
| **Depression** | 0 | 0 | 0 | 0 | 0 | 0 | 0 |
| **All cause mortality** | 1,154 | 3,977 | 12,069 | 19,156 | 42,703 | 113,126 | 192,185 |
|  |  |  |  |  |  |  |  |
| **DALY, Females** | **15-29** | **30-44** | **45-59** | **60-69** | **70-79** | **80+** | **Sum** |
| **Stroke** | 1,604 | 8,422 | 20,905 | 28,003 | 65,518 | 102,514 | 226,967 |
| **Ischemic heart disease** | 1,358 | 10,391 | 36,833 | 58,918 | 107,367 | 122,985 | 337,852 |
| **Other cardiovascular and circulatory diseases** | 2,250 | 8,250 | 17,269 | 23,649 | 45,300 | 52,835 | 149,553 |
| **Type-2 diabetes** | 33 | 6,991 | 12,065 | 11,506 | 14,637 | 11,707 | 56,938 |
| **Colon cancer** | 505 | 3,357 | 12,041 | 14,746 | 17,157 | 10,340 | 58,147 |
| **Breast cancer** | 1,031 | 28,796 | 69,958 | 48,848 | 34,597 | 18,527 | 201,757 |
| **Dementia and Alzheimer’s disease** | 119 | 220 | 5,104 | 11,835 | 37,819 | 96,059 | 151,156 |
| **Depression** | 48,476 | 68,637 | 58,617 | 26,380 | 17,661 | 11,135 | 230,906 |
| **All cause mortality** | 549,919 | 881,979 | 1,064,329 | 855,077 | 996,064 | 929,095 | 5,276,463 |
|  |  |  |  |  |  |  |  |
| **YLD, Females** | **15-29** | **30-44** | **45-59** | **60-69** | **70-79** | **80+** | **Sum** |
| **Stroke** | 245 | 955 | 2,952 | 4,810 | 9,021 | 9,173 | 27,156 |
| **Ischemic heart disease** | 476 | 2,756 | 7,150 | 7,749 | 8,630 | 6,832 | 33,593 |
| **Other cardiovascular and circulatory diseases** | 567 | 2,119 | 4,345 | 4,765 | 6,895 | 6,486 | 25,176 |
| **Type-2 diabetes** | 19 | 5,267 | 8,957 | 7,830 | 7,853 | 4,990 | 34,917 |
| **Colon cancer** | 11 | 84 | 382 | 588 | 906 | 1,007 | 2,979 |
| **Breast cancer** | 32 | 1,223 | 4,239 | 4,341 | 4,645 | 4,180 | 18,660 |
| **Dementia and Alzheimer’s disease** | 0 | 55 | 3,678 | 6,744 | 16,146 | 40,490 | 67,113 |
| **Depression** | 48,476 | 68,637 | 58,617 | 26,380 | 17,661 | 11,135 | 230,906 |
| **All cause mortality** | 477,220 | 694,196 | 658,601 | 415,504 | 383,314 | 302,601 | 2,931,436 |
|  |  |  |  |  |  |  |  |
| **YLL, Females** | **15-29** | **30-44** | **45-59** | **60-69** | **70-79** | **80+** | **Sum** |
| **Stroke** | 1,359 | 7,466 | 17,953 | 23,194 | 56,498 | 93,341 | 199,811 |
| **Ischemic heart disease** | 883 | 7,636 | 29,683 | 51,169 | 98,737 | 116,153 | 304,259 |
| **Other cardiovascular and circulatory diseases** | 1,683 | 6,132 | 12,924 | 18,884 | 38,406 | 46,349 | 124,377 |
| **Type-2 diabetes** | 14 | 1,723 | 3,108 | 3,676 | 6,783 | 6,717 | 22,021 |
| **Colon cancer** | 494 | 3,273 | 11,659 | 14,159 | 16,251 | 9,333 | 55,169 |
| **Breast cancer** | 999 | 27,573 | 65,719 | 44,507 | 29,952 | 14,347 | 183,097 |
| **Dementia and Alzheimer’s disease** | 119 | 165 | 1,426 | 5,091 | 21,672 | 55,569 | 84,042 |
| **Depression** | 0 | 0 | 0 | 0 | 0 | 0 | 0 |
| **All cause mortality** | 72,699 | 187,784 | 405,729 | 439,572 | 612,750 | 626,496 | 2,345,029 |

**Supplementary Table A.6:** Summary of key model inputs, their variability, and description of uncertainty/what if, analyses

| **Parameter** | **Analysis** | **Estimates** | **Description and references** |
| --- | --- | --- | --- |
| Over reporting of non-traffic related physical activities | Variability, triangular | Min:0 .5  Median:0.75  Max:1 | Sensitivity analysis assuming that people over report mean time spent in various non-travel related activities. Based on author judgment. |
| Variability in transferring the daily travel pattern data to weekly data (cycling) | Variability,  uniform | Min:2  Max: 2.3 | A greater % of people cycle in a week than in a day but we only have weekly data for England & Wales. Range is based on 1) ratio of daily diary to weekly diary from English & Welsh National Travel Survey and 2) daily to reported behaviour in a typical week. |
| Variability in transferring the daily travel pattern data to weekly data (walking) | Variability,  uniform | Min: 2.9  Max:3.1 | People who walk during a week may not walk every day. Range is based on 1) those who report walking more than one mile on any given day to those who report walking more than one mile in a given week both from diary data 2) any walking on one day to any walking in a typical week. |
| Relative risk for all cause mortality | Uncertainty,  normal | Mean:0.81 std:0.020 | RR per 8.6 mMETh per week ([Woodcock et al., 2011](#_ENREF_11)). Recalculated from RR 0.81 and METh 11.25. |
|  |  |  | Wen et al., 2011, recalculated as marginal METs  Inactive 0 > 3.75 mMETh/wk RR 1; low activity 3.75 > 7.5 mMETh/wk RR 0.86; moderate activity 7. 5 > 16.5 mMETh/wk RR 0.8; high activity 16.5 > 25.5 mMETh/wk RR 0.71; very high activity >=25.5 mMETh/wk RR 0.65 |
| Power transformation of the dose-response function | Uncertainty,  triangular | Min:0.25  Mode:0.5  Max:1 | Power transformation used in first degree fractional polynomial dose-response function (see figure B.1). Power transformation defines the shape of the non-linear dose-response function. See Woodcock et al. ([2011](#_ENREF_11)) for details. (See figure B.1) |

**Appendix B: Supplementary figures**

**Figure B.1**: Dose-response curves for dementia (upper graph) and for stroke (lower graph) for males. The lines represent alternative shapes of dose-response curves. The shape of the curve varied between iterations so that the exact shape was considered to be uncertain. Power transformation is the exponential function that defines the linearity. With a value of 1 dose-response is linear, with the value of 0.25 it is most curved.


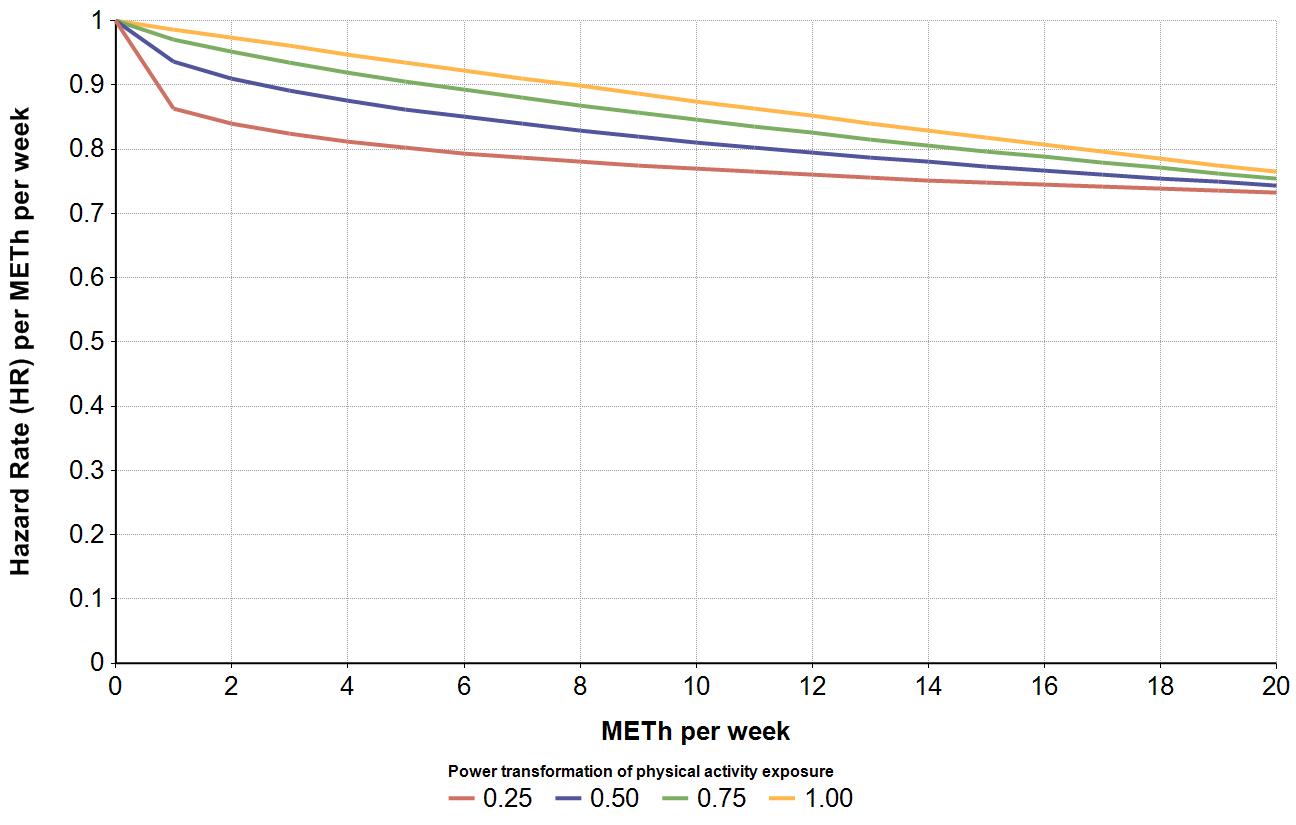


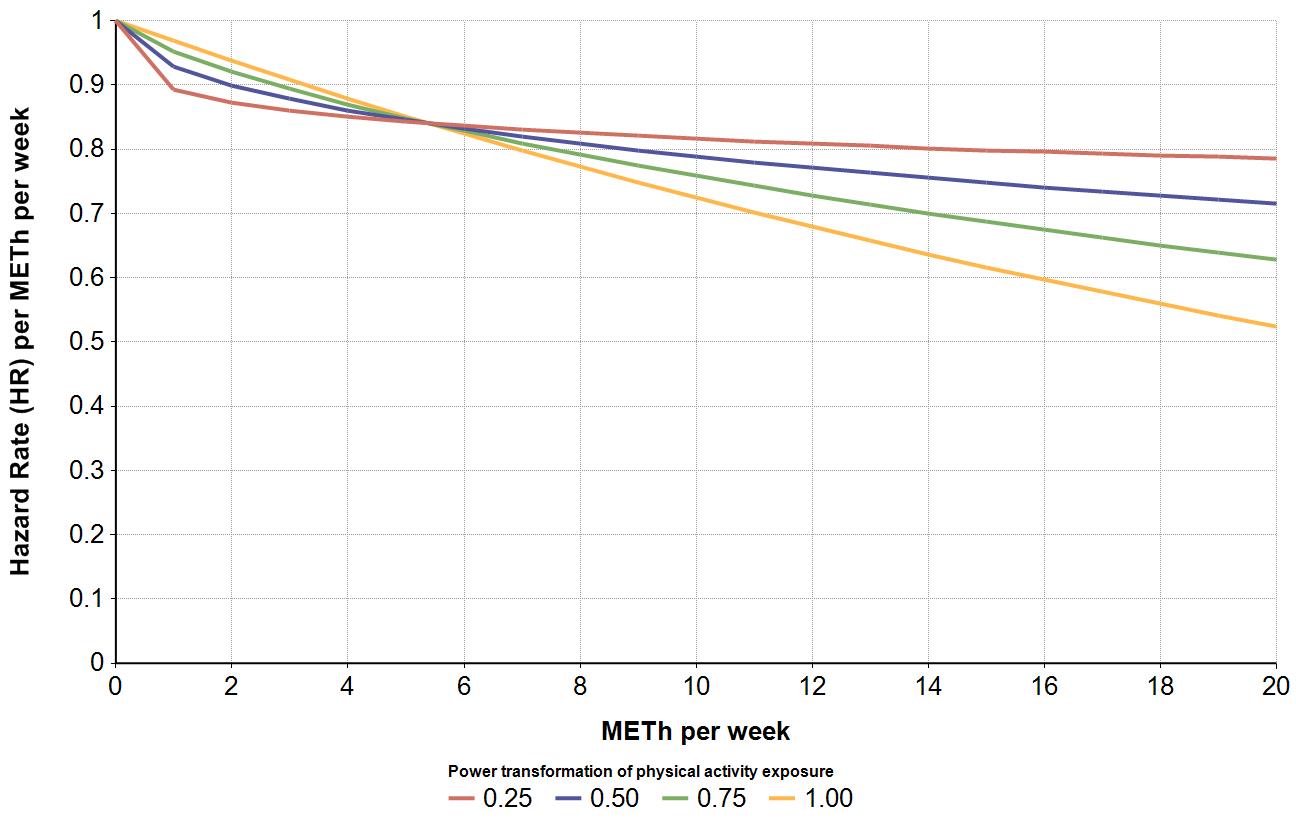


**Figures B.2:** Tornado plots from sensitivity analyses.

Tornado plots illustrate high and low model estimates from substituting the parameter of interest with the 97.5^th^ percentile of the parameter distribution within each age-gender stratum, and with the 2.5^th^ percentile, respectively. For example, for the marginal METs estimate for walking of a mean of 2.5 METs and standard deviation of 1.6 METs (lognormal distribution)(see Table A.2), sensitivity analysis would use a low estimate of 0.7 METs (2.5^th^ percentile) and a high estimate of 6.6 METs (97.5^th^ percentile). All the input parameters included in sensitivity analysis are presented in Table A.2 and A.6.


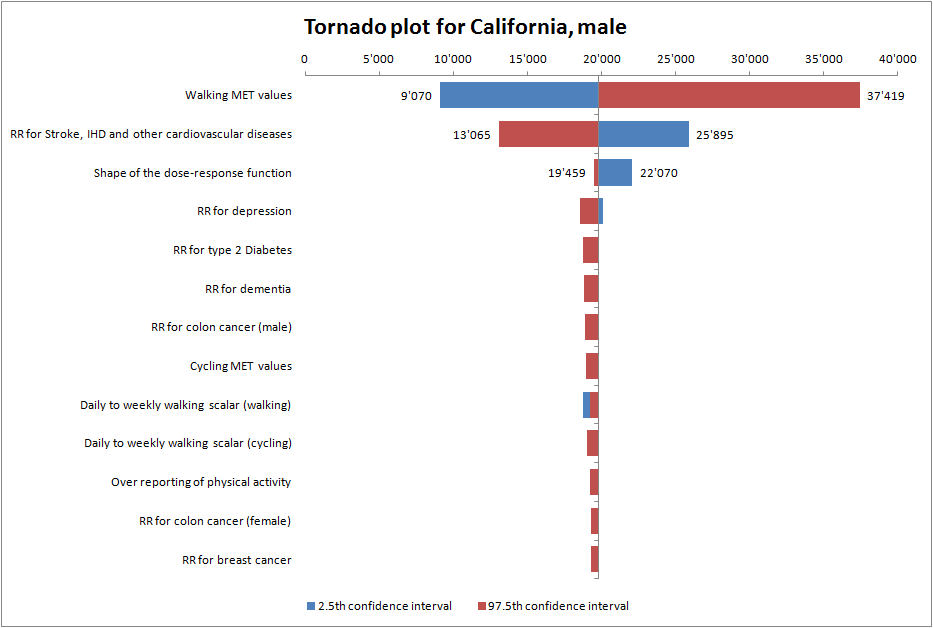


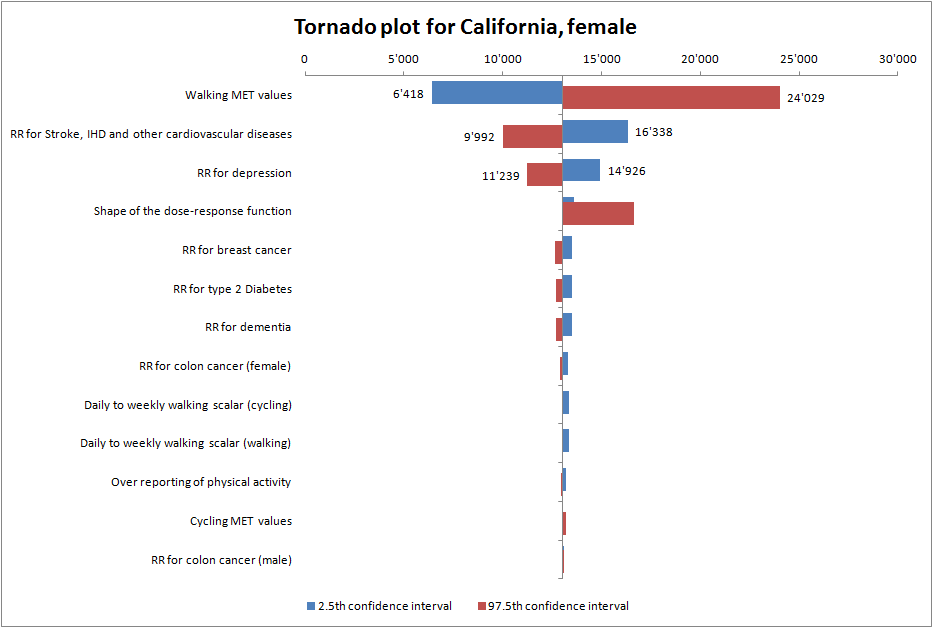


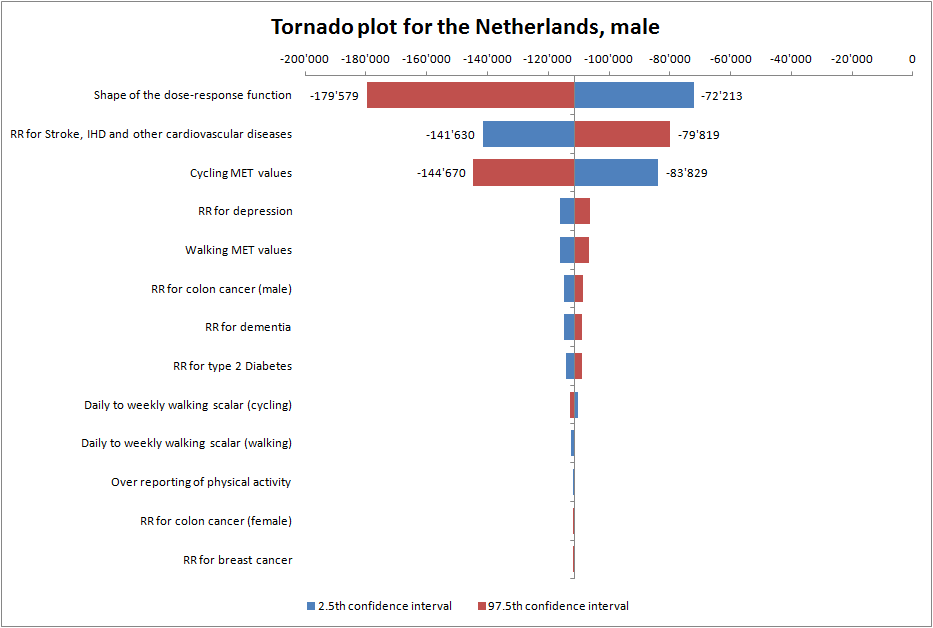

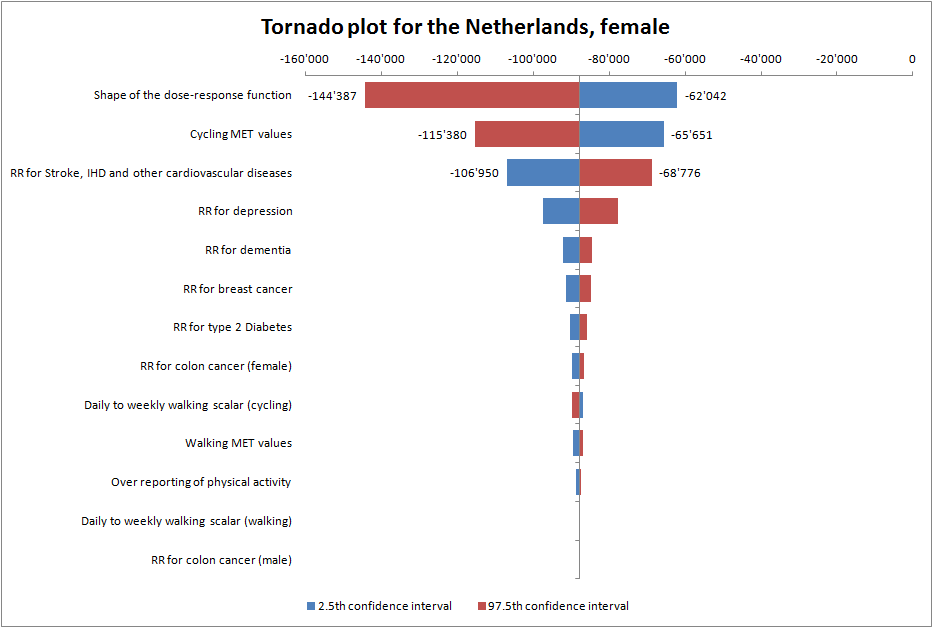


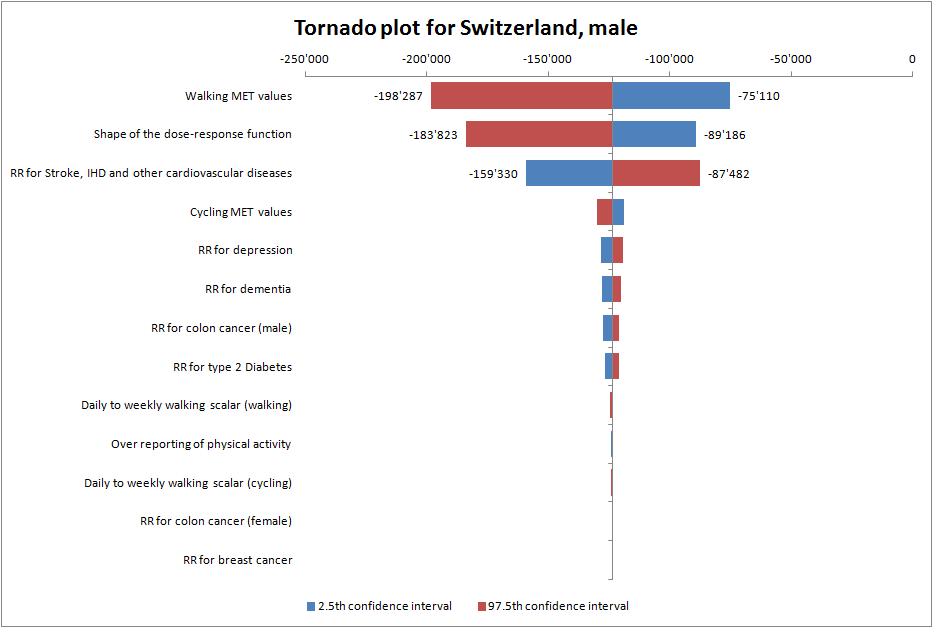

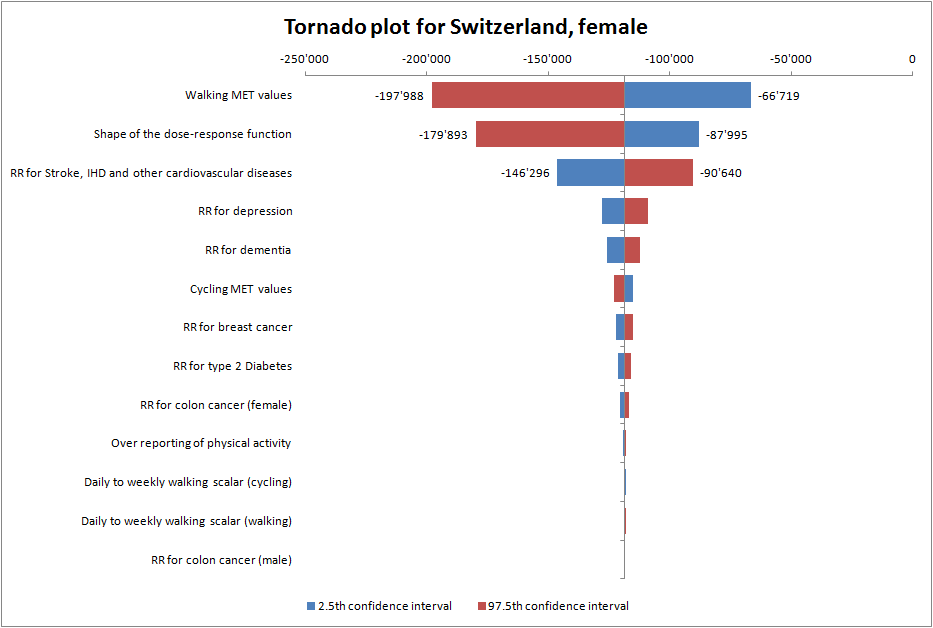


**Figure B.3 Gender distribution of walking and cycling in England &Wales and comparison areas.**

**Appendix C: References**

Ainsworth, B.E., Haskell, W.L., Herrmann, S.D., Meckes, N., Bassett, D.R., Jr., Tudor-Locke, C., Greer, J.L., Vezina, J., Whitt-Glover, M.C., et al., 2011. 2011 Compendium of Physical Activities: a second update of codes and MET values. Med Sci Sports Exerc 43:1575-81.

Ainsworth, B.E., Haskell, W.L., Whitt, M.C., Irwin, M.L., Swartz, A.M., Strath, S.J., O,Brien, W.L., Bassett, D.R., Jr., Schmitz, K.H., et al., 2000. Compendium of physical activities: an update of activity codes and MET intensities. Med Sci Sports Exerc 32:S498-504.

Bundesamt für Statistik, Bundesamt für Raumentwicklung, Ecoplan, 2007. Mobilität in der Schweiz: Ergebnisse des Mikrozensus 2005 zum Verkehrsverhalten. Bundesamt fuer Statistik, Neuchatel.

Craig, R., Mindell, J., 2013. Health Survey for England 2012, (HSE). The Health and Social Care Information Centre, London.

Department of Transport, 2013. National Travel Survey statistics, 7/30/2013 ed.

Federal Highway Administration, 2010. National Household Travel Survey. US Department of Transportation.

IHME, 2013. Global Burden of Disease Study 2010, in: United Kingdom Global Burden of Disease Study 2010 (GBD 2010) (Ed.), Results 1990-2010. Institute for Health Metrics and Evaluation (IHME), Seattle, United States.

Ministerie van Verkeer en Waterstaat, 2010. "Onderzoek Verplaatsingen in Nederland" (OViN).

Wen, C.P., Wai, J.P.M., Tsai, M.K., Yang, Y.C., Cheng, T.Y.D., Lee, M.-C., Chan, H.T., Tsao, C.K., Tsai, S.P., et al., 2011. Minimum amount of physical activity for reduced mortality and extended life expectancy: a prospective cohort study. The Lancet 378:1244-53.

Woodcock, J., Edwards, P., Tonne, C., Armstrong, B.G., Ashiru, O., Banister, D., Beevers, S., Chalabi, Z., Chowdhury, Z., et al., 2009. Public health benefits of strategies to reduce greenhouse-gas emissions: urban land transport. Lancet 374:1930-43.

Woodcock, J., Franco, O.H., Orsini, N., Roberts, I., 2011. Non-vigorous physical activity and all-cause mortality: systematic review and meta-analysis of cohort studies. Int J Epidemiol 40:121-38.
